# Thirty years with cervical vestibular myogenic potentials: a critical review on its origin

**DOI:** 10.3389/fneur.2024.1502093

**Published:** 2025-01-22

**Authors:** Jonas Bruun Kjærsgaard, Dan Dupont Hougaard, Herman Kingma

**Affiliations:** ^1^Department of Otolaryngology, Head & Neck Surgery and Audiology, Aalborg University Hospital, Aalborg, Denmark; ^2^Department of Clinical Medicine, Aalborg University, Aalborg, Denmark

**Keywords:** cVEMP, vestibular evoked myogenic potentials (VEMP), vestibulocollic reflex, sacculus, utriculus, specificity, semicircular canal (SCC), afferent fiber

## Abstract

Myogenic potentials generated by acoustic stimulation of the vestibular system have been reported since 1964. This examination became better known as cervical vestibular evoked myogenic potentials (cVEMPs) and gained increasing clinical application since the nineties. Since its discovery, the saccule has been conceived as the most likely vestibular end-organ driving these myogenic potentials of the neck. As findings from both animal and human studies for a long time uniformly provided evidence supporting this theory, cVEMP assessment has become synonymous with evaluation of saccular and inferior vestibular nerve function. This review of the basic evidence supporting this conclusion, questions if cVEMP may be considered as being predominantly or even exclusively driven by the activation of any single vestibular end-organ. We conclude that the results of this review show that contributions from the crista ampullaris of all three ipsilateral semicircular canals, as well as the ipsilateral utricle cannot be ruled out in clinically conducted cVEMP assessments.

## Introduction

The vestibulocollic reflex (VCR) plays an essential role in head- and gaze stabilization and postural control during everyday life activities. For example, during natural human locomotion, such as walking, the VCR causes precise head movements in the pitch plane compensatory to the trunk movements in the same plane to preserve optimal spatial orientation and gaze stabilzation (1–4). For an adequate VCR in the yaw-, roll-, and pitch planes, an exact detection of the six degree of freedom head movements is required: three dimensions of rotation and three dimensions of translation.

At present, the integrity of the VCR can be assessed, at least partly, via cervical vestibular evoked myogenic potentials (cVEMP) elicited in the neck muscles and induced by high intensity acoustic stimulation of the labyrinth (> 90 dB sound pressure level). A precontracted sternocleidomastoid muscle (SCM) shows a decrease of the electromyogenic potential with sound stimuli and produce a positive peak with a latency of 13 milliseconds (p13) and a negative peak with a latency of 23 milliseconds (n23). Because the p13 amplitude is proportional to the degree of muscle contraction, cVEMP is absent without sufficient neck muscle contraction. CVEMP outcome parameters include amplitude, latency, asymmetry, and threshold, as described in detail in Rosengren et al. (5). However, in clinical practice different equipment and electrode placements are still used, complicating a straightforward comparison of the outcomes. Currently, the cVEMP is promoted as a routine clinical test to monitor the integrity of the saccular and inferior vestibular nerve function (6). CVEMP acquisition is, however, no easy routine test, as it requires extensive technical training. Inadequate technical training can lead to ambiguous cVEMP outcomes. Also, measuring and interpretation of cVEMP in clinical practice is not “plug and play.” as it is often difficult to identify the typical response pattern near the response threshold. Furthermore, reproducibility at suprathreshold, can be a challenge as the p13 amplitude may be affected by muscle fatigue or atrophy (especially important in the elderly population). Consequently, clinical test results may be ambiguous. Unilateral and bilateral absent or severely reduced responses are often seen with increasing age, where a reduced ability to produce a sustained muscle contraction or muscle atrophy may act as a confounding factor (7–10). Another complicating issue is that in some patients no unilateral cVEMP responses are found, but at follow-up a cVEMP response can be demonstrated (11). This may point toward an unlikely restorage of function or instead indicates that cVEMP testing and interpretation is not so clear-cut and straight forward and that there is an inherent risk of misinterpretation and -classification (7–10). This uncertainty is partly accounted for, as abnormal cVEMP test results are only part of the diagnostic criteria for superior canal dehiscence syndrome (SCDS), as illustrated by the international diagnostic guidelines (12–16). With SCDS, the typical case history, the significantly decreased cVEMP thresholds and the visualization of the dehiscence with a high-resolution CT scan may be used as exclusive diagnostic criteria. Despite of this, clinical usage of cVEMP is encouraged in multiple reviews and at many conferences, as cVEMP testing has been shown to be absent or reduced in a host of vestibular- and neurological disorders (5, 17–19). This contrast is peculiar and raises not only the question to which degree there is evidence to merit routine cVEMP testing in vestibular clinics in general, but also to which certainty the origin of cVEMP is delineated.

We decided to critically review the extensive literature to identify the current gaps in our understanding regarding the precise nature of cVEMP given the current state-of-the-art knowledge. In theory, it could be that cVEMP selectively reflect the saccular contribution to the VCR. Logically, however, if cVEMP are to be attributed to the function of the saccule, it must either (1) stem from a selective activation the saccule or (2) the specific projections of the possible co-activated end-organs must be so weak that their contributions to the cVEMP are indisputably negligible. Below we will argue that it is more likely that the cVEMP, like the VCR, is influenced by the input of multiple, possibly all, ipsilateral vestibular end-organs.

## The discovery of cervical vestibular myogenic potentials and its early development

**Figure 1:**
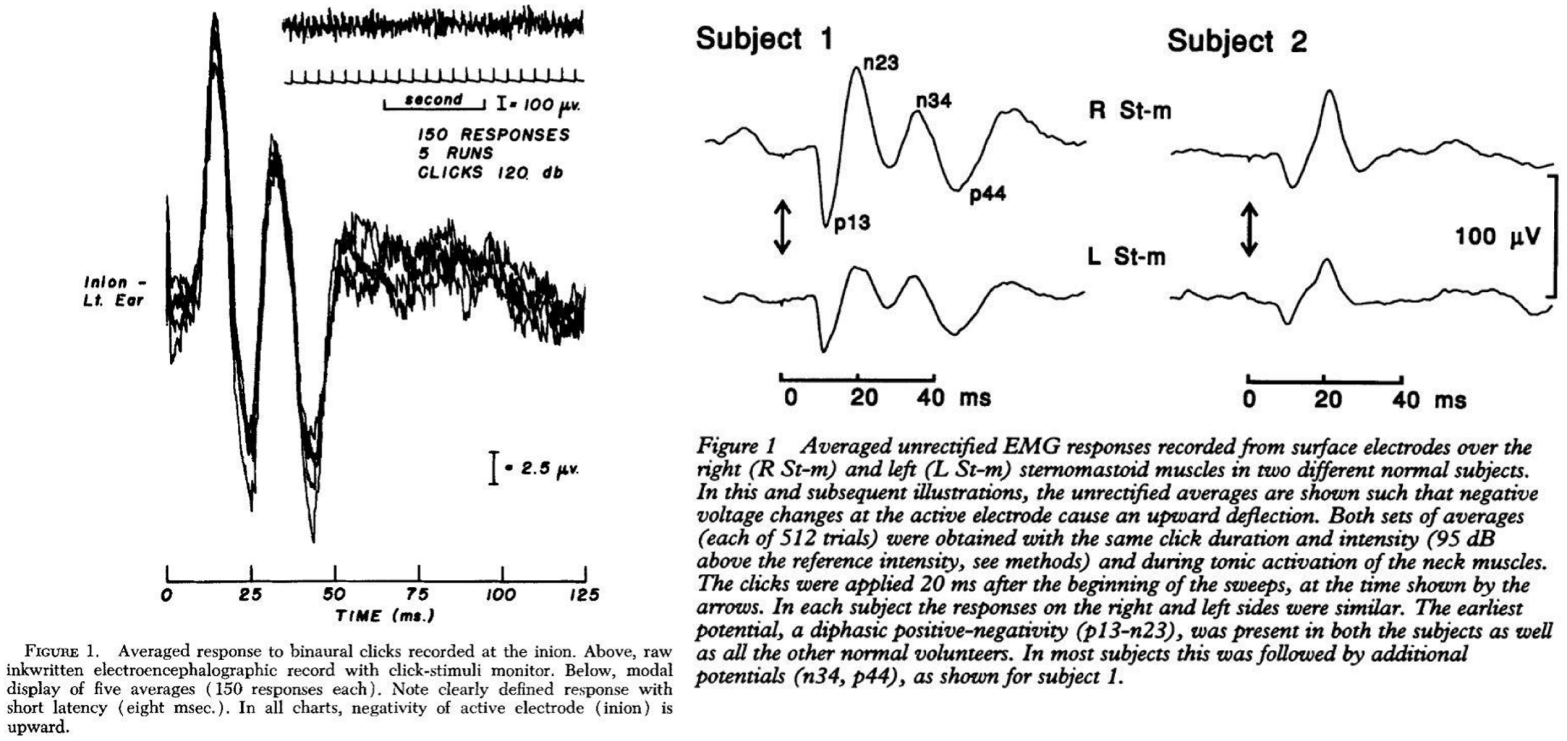
Electrographic recordings from the inion and sternocleidomastoid muscle after acoustic stimulation. Left Cody et al. 1964 (20), Right Colebatch et al. 1994 (21).

The earliest discovery of cervical myogenic responses to loud air-conducted sound (ACS) stimuli were, to our knowledge, presented by Bickford, Jacobson, and Cody in 1964 (20). In their extensive and novel studies on humans they were able to elicit a short latency myogenic potential at the inion (external occipital protuberance) with methods strikingly similar to how clinical cVEMP testing is conducted today. In 1994, 30 years later, Colebatch and Halmagyi confirmed their results (21). However, with their experiments, the position of the active electrodes was directly placed on the skin above the SCM and not on the inion. They reported the loss of this reflex in relation to selective vestibular nerve section (21, 22). As already indicated above, the SCM response was characterized by a positive peak 13 milliseconds and a negative peak 23 milliseconds after the stimulus onset (collectively termed p13n23), similar to the inion response in latency but with an inversion on polarity, as shown in Figure 1 (21, 22). The examination of the short latency SCM responses to acoustic stimulation were conceptualized as ‘VEMP’ in 1996 (23). During the decade that followed the conceptuality of ACS cVEMP, subsequent studies found that both galvanic stimulation, bone-conducted vibration (BCV) and reflex hammer taps on the temporal- or frontal bone were also able to generate an equal response (10, 24–27). Consequently, the concept of ‘VEMP’ had to be expanded to include these types of stimuli. The prefix ‘c’, denoting cervical was added when a similar, yet inversed, ocular muscle response to acoustic stimulation was discovered (28).

In their original study, Bickford et al. compared the responsiveness to sound in patients with and without intact horizontal semicircular canal (SCC) function, as measured with cold water caloric stimulation (20). In their subsequent studies on patients with vestibulopathies, it was reported that, in one patient who was treated with streptomycin who had absent responses to cold water caloric stimulation, sound induced inion responses could still be elicited (29). Considering these findings and based upon the observations by McGee et al. in guinea pigs, that streptomycin preferably damages hair cells of the crista ampullaris and less the otolith organs, Townsend and Cody found it unlikely that the SCCs were the receptor of the inion response (29, 30). When Townsend and Cody furthermore found the inion response to be absent in 10 out of 22 ears with endolymphatic hydrops, which they perceived to predominantly affect the saccule over the utricle, they, by triangulating evidence, concluded that stimulation of the saccule was most likely responsible for the inion response (29).

Similarly, from early on, the cVEMP response was assumed to be of saccular origin, as irregular afferents from the saccule in the squirrel monkey had previously been found to be the most sensitive fibers to ACS stimulation in comparison to the afferents from the other vestibular end-organs (31). The thought that the electromyogenic response to ACS originates from the saccular activation was further strengthened by the discovery of ACS responsive irregular fibers in both the guinea pig and the cat (32, 33). When the earliest studies in the guinea pig furthermore only showed a negligible activation of the afferents from the SCCs and utricle, cVEMP in humans was considered also to originate from the saccule through direct extrapolation of animal research (24, 33). Since then, despite the limited direct evidence, cVEMP has been promoted as a potential clinical test of saccular function.

Evidently, the scientific focus on allocating the vestibular myogenic responses to one single vestibular end-organ has a long tradition of using results from patients with more or less well-defined lesions within the inner ear (22, 29). However, it would be remarkable if the cVEMP, being a constituent of the VCR, depends exclusively or predominantly on saccular input, as the VCR requires input from all labyrinthine organs to execute its function in head stabilization (4, 21, 34). Already, in 2010, Welgampola and Carey asked for more exclusive evidence for the origin of cVEMP, before the saccular predominance theory could be generally accepted (35). This uncertainty of the origin is also mentioned in ‘The Vestibular System’ by Goldberg et al. (36). The growing skepticism with regards to the exact clinical relevance of the cVEMP in clinical practice encouraged us to investigate the current state of the art knowledge regarding the origin of the cVEMP in detail.

## An investigation of origin

### Responsiveness of vestibular afferents

The complexity of understanding the nature of the vestibular sensitivity to sound or vibration within the audible frequencies is reflected by the numerous animal studies, the variety of animal models used, and their findings (31–33, 37–46). A list of these animal studies, the methods used, and conclusions are summarized in Table 1. In general, the animal studies were conducted by singling out vestibular afferents, allocating it to a specific end-organ and then examining their responsiveness to different intensities of ACS or BCV (31). An important reconciliation between conflicting findings in the same animal model was given by Curthoys et al. in 2012, and further explained in their paper from 2016 (39, 46). Here, Curthoys et al. concluded that the reported insensitivity of utricular afferents to ACS in their previous studies was due to experimental limitations, as his research group later managed to activate the utricular afferents by ACS when verifying their results (33, 37, 39, 44).

**Table 1:** Animal studies whose primary outcome measures included exploration of the natural responsiveness of vestibular afferents to air conducted sound (ACS) and bone-conducted vibration (BCV).

Study	Animal model	Stimulus characteristics	Recording site	Author conclusions
Young et al., 1977 (31)	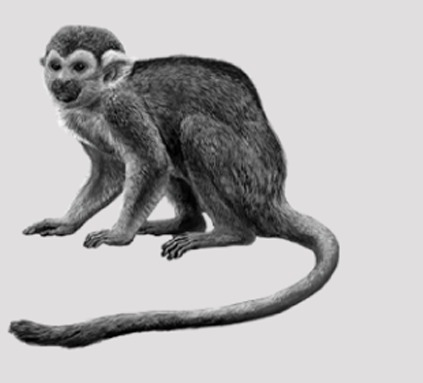	ACS and BCV—Sustained pure tones (3 s)	Proximal (common) VN	Afferents from all five end-organs are responsive to both ACS and BCV at high intensities. Saccular afferents are the most sensitive to ACS. No differential activation using BCV. Irregular fibers are more easily activated by acoustic stimulation than regular fibers
McCue & Guinan, 1994 (32)	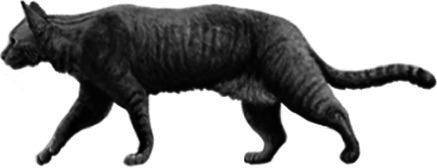	ACS—clicks (0.1 msec) and 800 Hz TB (50 msec)	Only IVN, near the saccular tributary	Irregular fibers originating from the saccule are responsive to moderate intensity acoustic stimulation
Murofushi & Curthoys, 1997 (33)†	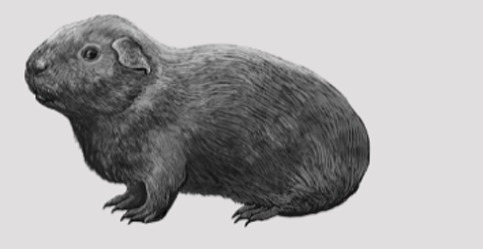	ACS—clicks (0.1 msec)	Scarpa’s ganglion, not further specified	Irregular afferents originating from the saccule is responsive to moderate intensity acoustic stimulation. No evidence of activation of afferents originating from the utricle or SCCs.
Curthoys et al., 2006 (37)†	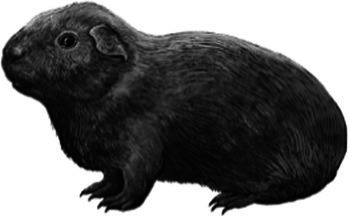	ACS and BCV—clicks, pure tones of unspecified duration and 500 Hz TB (7 msec)	Scarpa’s ganglion, preferably sampling the superior vestibular nerve division	Most irregular afferents originating from the otoliths are activated with low intensity BCV. None of the BCV-activated fibers responded to ACS. Only few irregular afferents originating from SCC are activated by BCV.
Curthoys et al., 2012 (46)	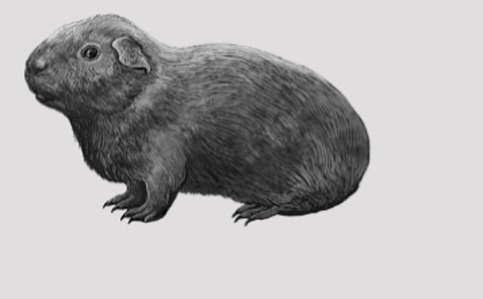	ACS and BCV—500 Hz pure tones (0.5 s)	Scarpa’s ganglion, sampling utricular and saccular afferents	Utricular and saccular irregular afferents are both responsive to ACS and BCV.
Zhu et al., 2014 (38)	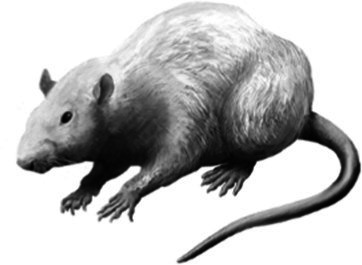	ACS—clicks (0.1, 0.3, 0.5 and 1 msec)	Both SVN and IVN	Irregular afferents from all five end-organs are responsive to ACS, well within the intensities used for clinical cVEMP. At high intensity there is a considerable activation of afferents from both the anterior SCC and the otoliths.
Curthoys et al., 2016 (39)*	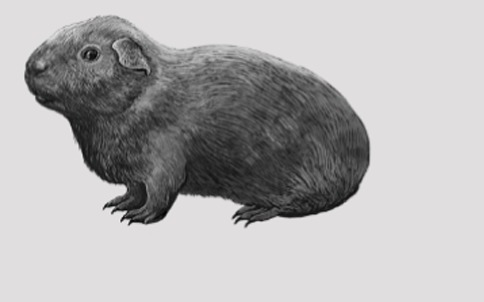	ACS and BCV—TB at multiple frequencies.	Scarpa’s ganglion, throughout sampled	Utricular and saccular irregular afferents are responsive to ACS. The mean threshold for saccular afferents were 10-15 dB below that of utricular ones. All ACS responsive afferents were also BCV sensitive.
Huang et al., 2022 (40)	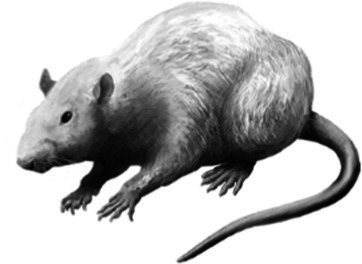	ACS—TB at multiple frequencies, duration 10 msec, with 1 msec ramping	Both SVN and IVN	Irregular afferents from all five end-organs are responsive to ACS. At ACS intensities that strongly activates otolithic afferents a considerable number of canal afferents is activated.

VN, Vestibular nerve; IVN, inferior vestibular nerve; SVN, superior vestibular nerve; TB, Tone-burst. Denotations; †related to reconciliation by Curthoys et al. (39). *Experimental data likely overlapping with Curthoys et al. (46). Gray-scaled animal figures are from TogoTV (© 2016 DBCLS TogoTV, CC-BY-4).

In summary, multiple animal studies now unanimously provide evidence that the utricular and saccular afferents are equally sensitive to BCV (31, 37, 39). However, when using ACS, there appears to be a difference in sensitivity between afferents originating from each otolith organ, as findings in one animal model show utricular afferents to be, on average, 10-15 dB less sensitive than saccular afferents (39). Nonetheless, the spread of thresholds among individual saccular afferents overlaps substantially with the thresholds of their utricular counterparts. This activation overlap, that reduces the possibility for a clear selective saccular activation, has consistently been confirmed in another animal model (38, 40, 43). Therefore, with increasing intensities, more afferents from both the saccule and the utricle will, irrespective of a difference in thresholds, be recruited and with near saturation stimulus intensity all sound sensitive afferents will be activated. The saturation of both saccular and utricular afferents activation in animal models appears to occur near 120-130 dB peak sound pressure level, which is equivalent to the ACS intensities used in clinical cVEMP setups (21, 38, 40). Additionally, more recent animal studies use clicks and tone-bursts equal to the ones used in clinical cVEMP testing, increasing their translational validity. These recent animal studies, furthermore, also clearly indicate that ACS intensities, that strongly activate afferents from the otolith organs, also activate afferents from all three SCCs (38, 40). These findings align with Young et al. who described ACS responsiveness of the SCC already in 1977, but on the other hand conflicts with the findings by Curthoys et al. (31, 39, 41).

**Figure 2:**
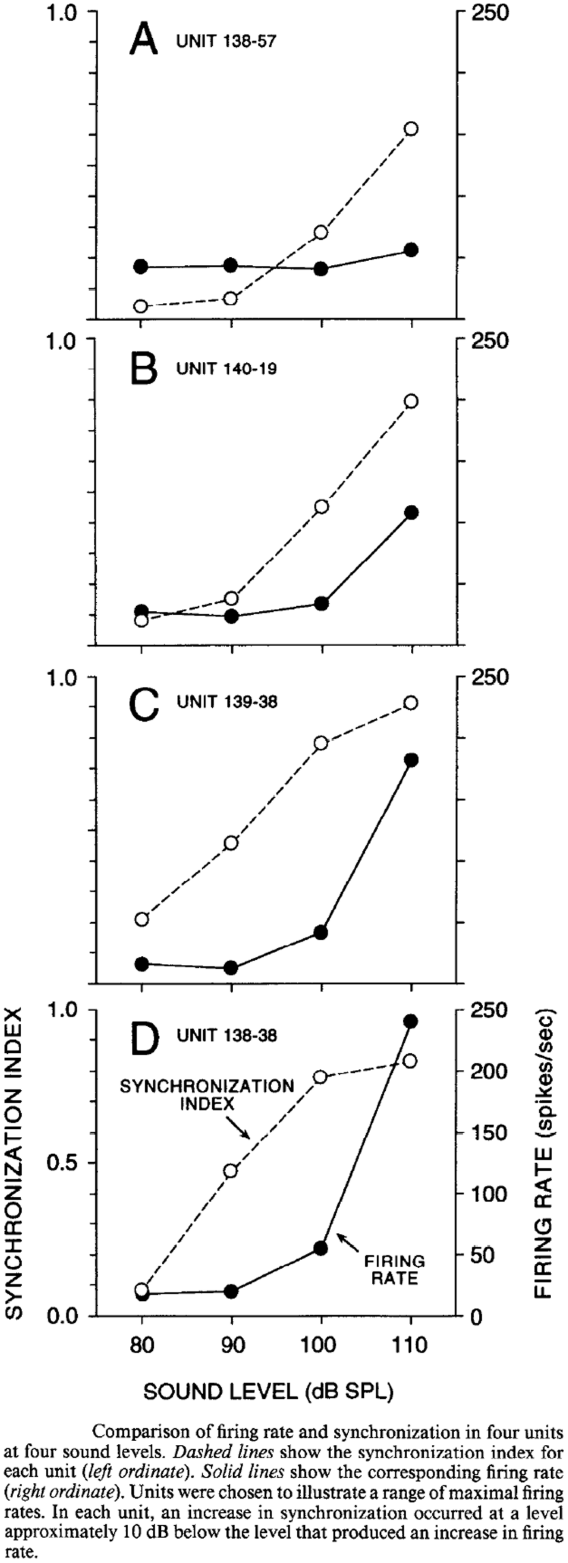
Comparison of synchronization vs. firing rate of single afferents at different stimulus intensities, McCue and Guinan 1994 (32).

**Figure 3:**
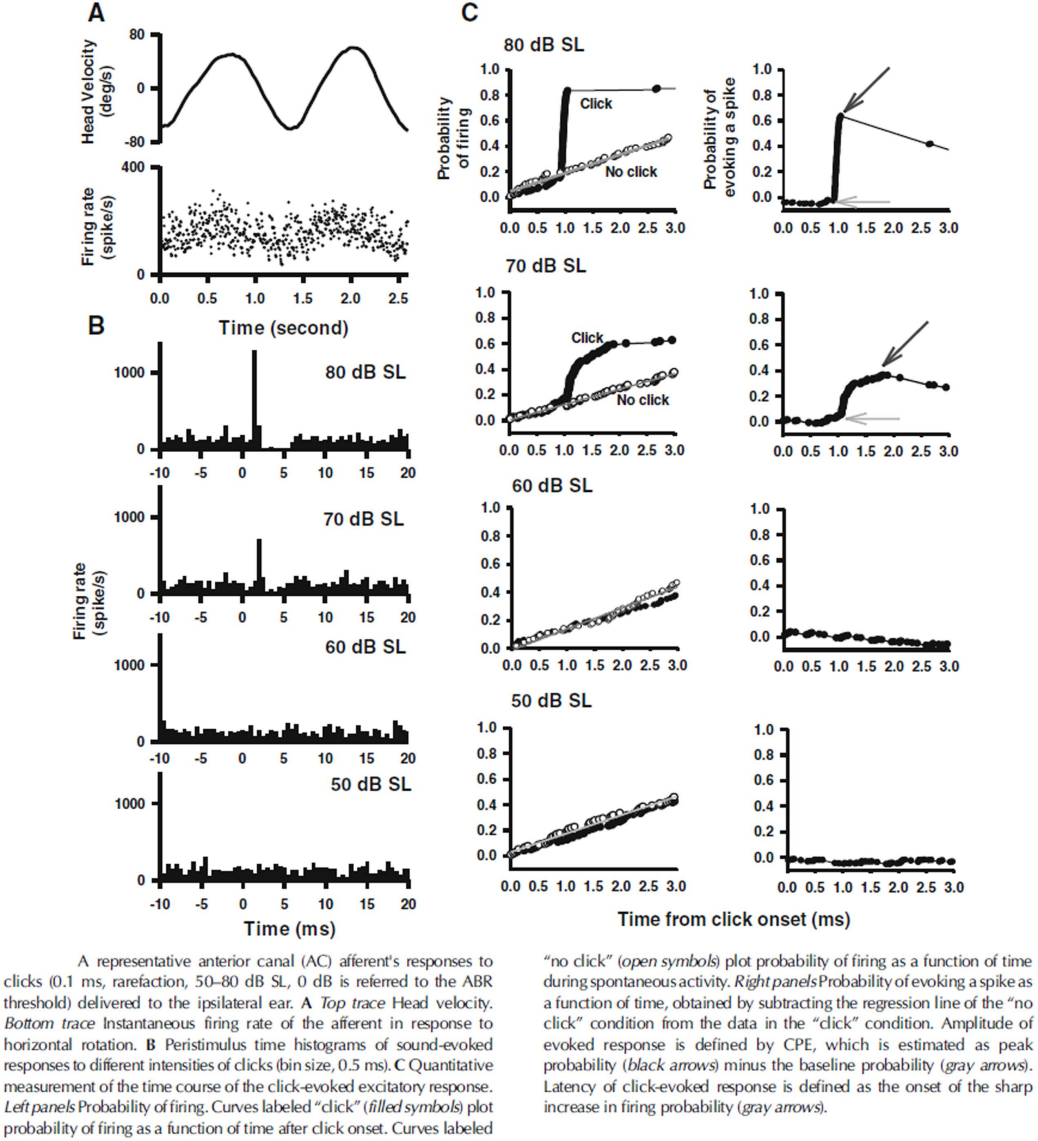
Response pattern of an anterior canal afferent after clicks at different intensities, from Zhu et al. (38). SL, denotes sensational level, and 50 dB is approximately equivalent to 100 dB peak SPL. CPE, denotes cumulative probability of evoking a spike.

A potential explanation for this discrepancy is the different criteria that have been used to determine afferent fiber activation. Curthoys et al. determined the sensitivity of afferent fibers based on a predefined significant rate-change of firing as a function of the intensity of a continuous sound stimulus. By that they found that macular afferents were more sensitive than SCC afferents, which underlines the interpretation that VEMPs are predominantly of utricular and/or saccular origin. However, another way to analyze the sensitivity of afferents to sound is by detecting modulation of the spike frequency phase-locked to the stimulus frequency. By calculating the Fourier spectrum of the response, which works as a kind of averaging technique, the signal-to-noise ratio of the threshold detection increases and the sensitivity to low intensity stimuli can be measured more accurately. McCue and Guinan in 1994 used both criteria when examining the fibers in saccular tributary of the inferior vestibular nerve (32). Here they reported, when using the more sensitive technique, that many afferents are indeed stimulated at sound intensities far below the thresholds reported by Curthoys (32). Figure 2 illustrates the different type of response to ACS, depolarization synchronization versus absolute firing rate, in vestibular fibers (32). Another technique to determine the sensitivity of afferents to sounds was established by measuring the latency of the generation of action potentials after a click or tone burst and calculating the probability of firing after stimulus onset (38, 40, 43). This technique “accumulates or averages” the responses to multiple clicks which also increases the signal to noise ratio to determine the sensitivity to sound. By this sensitive technique, it was shown that the SCC afferents are also sensitive to sound clicks as used in VEMP-testing (38, 43). The response pattern of a single irregular anterior canal fiber to click of various ACS intensities from Zhu et al. is shown in Figure 3 (38).

In summary, SCC afferents seem to be less sensitive to sound stimulation than macular afferents. However, it is likely that the extreme high intensity clicks, or tone-bursts used for clinical VEMP testing, depolarize afferents from multiple vestibular end-organs including the ones from the cristae ampullaris. It might be that, in humans, the macular activation is already fully saturated at the higher intensity stimuli used with clinical testing and the SCC activation is not, as recent animal studies indicate (38, 40). This finding of course requires further experimental confirmation.

### Neuronal circuity: can cVEMP be generated by utricular/saccular and/or semicircular canal activation?

**Figure 4:**
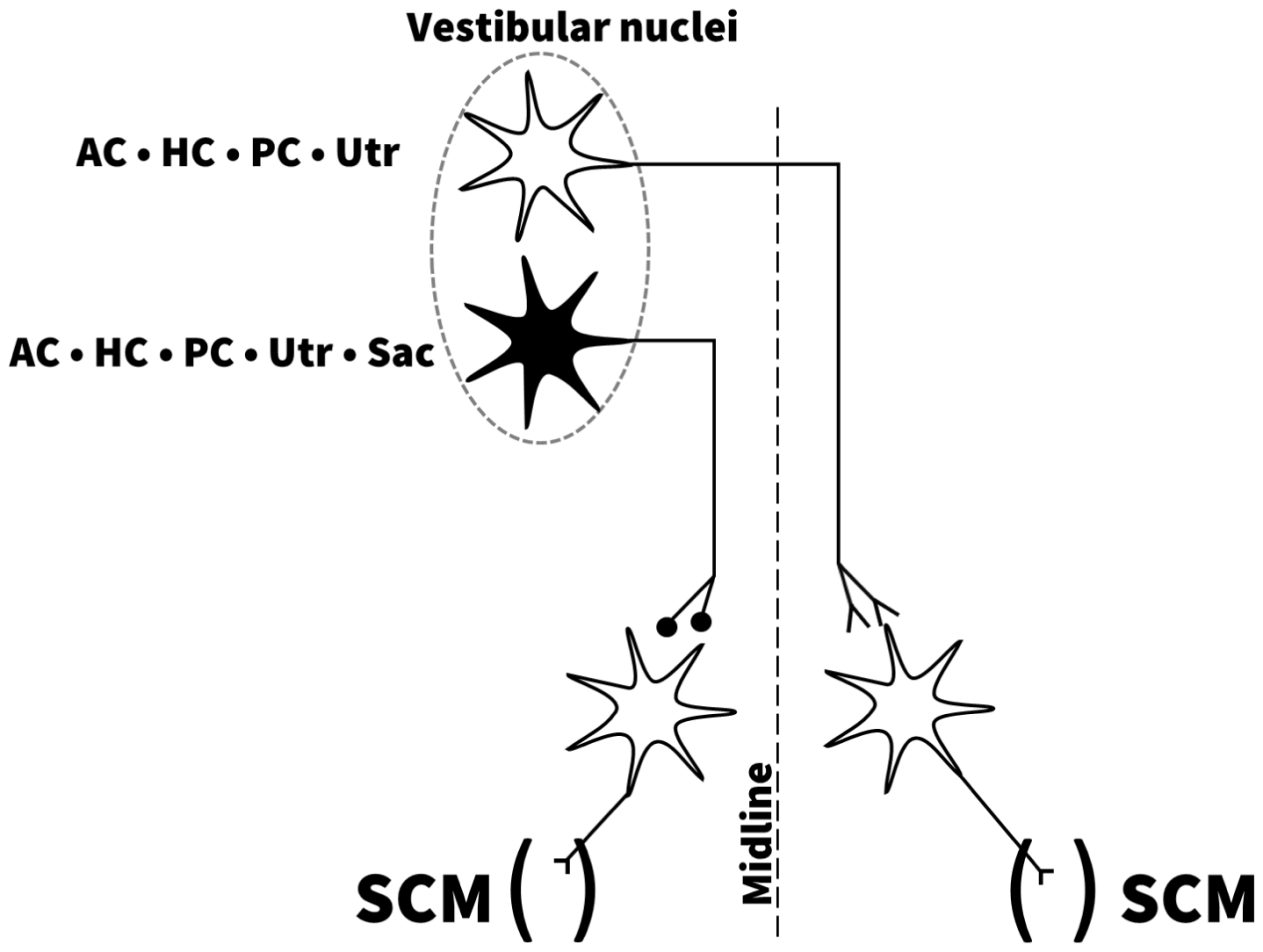
Independent projections from all five vestibular end-organs to the sternocleidomastoid muscles. Drawing based on description and illustration by Uchino et al. (53). AC; anterior semicircular canal, HC; horizontal semicircular canal, PC; posterior semicircular canal, Utr; Utricle, Sac; Saccule, SCM; sternocleidomastoid muscle.

Animal studies have shed light on the central neuronal circuity of the vestibular system in mammals, more precisely the contribution from the otolith organs to the VCR. Early studies in the cat have shown that selective electrical stimulation of the nerves from each of the SCCs produced a bilateral response on the SCMs of opposite directions, with a short latency inhibitory postsynaptic potential of the ipsilateral SCM (34). Direct stimulation of the utricular nerve demonstrated a short latency ipsilateral inhibition of the SCM and an inversed response of the contralateral SCM, analogous to SCC nerve activation (47). Electrical saccular nerve stimulation produced an ipsilateral inhibition of the SCM, but no response on the contralateral SCM (47). These projections are illustrated in Figure 4. These findings indicate that the utricle and SCCs have the necessary projections to produce a contralateral response of the SCM. As argued, they also show that all five vestibular end-organs independently have the necessary projections to produce an ipsilateral vestibular evoked myogenic potential on the SCM analog to the p13n23 (3, 34). The existence of similar projections in humans is supported by clinical research. In recent papers, in vestibular implant research, it has been confirmed it is possible to elicit cVEMPs by delivering a selective intralabyrinthine electrical stimulation at the level of all three ampulary nerves (48, 49). Naturally, the possibility of current spread from the ampullary stimulation to the otolithic organs cannot be entirely ruled out, but the focus on the stimulation is really on the ampullae here. This supports the hypothesis that cVEMP response can be mediated by the both the superior and inferior vestibular nerve. In contrast, Basta et al., using intraoperative direct electrical stimulation of the inferior and superior vestibular nerve in patients undergoing otosurgical procedures for vestibular schwannoma or neurovascular compression of the eight cranial nerve, were able to elicit ipsilateral SCM responses after stimulating the inferior nerve, but not the superior vestibular nerve (50). The lack of responses to superior vestibular nerve stimulation found by Basta et al. has to our knowledge not been reproduced nearly two decades later (50). We encourage further research on this topic, as it is unclear if the pathology, surgery, or experimental conditions themselves might have influenced the outcome of these studies (48–50).

### Neuronal processing: weighting of the contribution of utricular-, saccular- and semicircular canal activation of the VCR

The finding of a short-latency reflex from the otoliths to the ipsilateral SCM, as predicted in the original electrophysiological study on the inion response from 1964, provided a neuroanatomical framework to support the interpretation of ipsilateral cVEMP (47). However, the simple brainstem reflex, corresponding to a two or three synapse pathway, was quickly recognized as being inadequate to explain the dynamics of the VCR during natural vestibular stimulation (34). Therefore, further animal studies were undertaken to explore the central integration of these primary afferent signals (4, 51–57). Of particular importance for understanding early vestibular sensory integration, Uchino and colleagues in 2005 published a review on the neuroanatomical and physiological evidence for early vestibular sensory integration in mammals with the cat as animal model (53). They concluded that the majority of vestibular second order neurons receive afferent signals from more than one end-organ. These neurons, located in vestibular nuclei, may receive this converging input directly or through interneurons, often a single interneuron. If the input were polysynaptic, it could be either congruent or reciprocal (54).

The central vestibular integration has since been shown to be even more complex than just a convergence of primary afferents. In summary, ample evidence supports that second-order neurons within the vestibular nuclei receive and integrate sensory input in a highly complex manner: (1) The majority receive vestibular sensory signal originating from more than one end-organ, in any combination of SCCs and otoliths (52–54, 56), (2) They often receive both regular and irregular afferents, that are differentially activated, conveying distinct readings in two parallel complementing information systems (36, 52, 56, 57), (3) The sensory input is not processed centrally in a strict linear manner, but convergent afferent signals are weighted in a substantial portion of neurons (55). The specific weighting of the afferent input at a single neuronal level seems to be modulated by the synchronous input of other sensory afferents (56), and (4) Sensory input may be canceled out by extra-vestibular signaling, e.g., when mitigating the vestibular sensory activation that results from self-motion (reafference) (55, 57).

Animal studies furthermore illustrate that most of these integrating neurons in the vestibular nuclei have vestibulospinal projections terminating at the cervical level; often at SCM motorneurons (3, 47, 52, 54). This directly underlines the relevance of these findings to the cVEMP. The acknowledgement of this complex signal processing in the central vestibular system is vital to understanding the VCR in man, yet it has only more recently been put into the direct context of cVEMP in the comprehensive and reflective review by Corneil and Camp (7). However, they did not entirely emphasize what implications these findings should have on the widespread clinical interpretation of cVEMPs.

The complex convergence of afferents, and independent activation from each end-organ nerve to the ipsilateral SCM reveal that the cVEMP is likely to be a common pathway response. Recognition of a cVEMP-configuration therefore only reflects that some part of the vestibular system has been activated. The input convergence and common pathway in the efferent part of the reflex makes changes following peripheral or central lesions difficult to predict, especially if central compensation within the vestibular nuclei has occurred. It is, therefore, not unreasonable to think that only small changes in cVEMP may be expected even with complete loss of sensory capacity of a single end-organ, given that another end-organ may also be strongly activated (36, 54). On the other hand, incomplete lesions affecting multiple end-organs may possibly abolish the reflex despite no end-organ is singly severely impaired. This is possibly demonstrated in the findings in patients with acute vestibular neuritis by Taylor et al. who reported low degree of concordance between function of the posterior SCC and cVEMP findings (58). In 50% (8/16) of their patients with abnormal cVEMP assessments the results instead coincided with evidence of the superior vestibular nerve being affected. Six of these displayed abnormal function of the anterior and horizontal SCCs and an abnormal ocular VEMP.

The weighing effect between primary afferent input and activation at second-order neuron level precludes simple linearity inferences to be drawn, especially across different end-organs. This implies for example that even though the anterior and horizontal SCCs are only moderately activated their contribution to the cVEMP could be substantial, and certainly cannot be ruled out at present (7, 37, 38, 40, 43). A prerequisite for claiming a negligible contribution from the SCCs would be specific evidence on the weighing of primary afferent input at a central level from well-designed lesion-studies. To the best of our knowledge, such evidence is not available at present.

An existence of cervical projections with differential strength between the utricle and saccule to the ipsilateral SCM is implied by in multiple reviews, consistently referencing the review by Uchino et al. from 2011 (3, 18, 59, 60). In two reviews, this is explicitly claimed (17, 61). But to the knowledge of the authors, there is no direct evidence that the saccular projection to the ipsilateral SCM should be any stronger than the utricular one. Neither in the original animal study from 1999 nor the work of 2011 (3, 47). Furthermore, Uchino, Kushiro and their colleagues never speculated on differences in strengths of the projections to the neck muscles. If one scrutinizes the findings of the original animal study, the activation of the SCM motoneurons by electrical stimulation at the saccular and utricular nerve level, did show that one of the nerves was more easily activated than the other (47). However, the most sensitive nerve was found to be the utricular one. More specifically, to produce a response in SCM motoneurons utricular nerves required a mean electrical current of 11.4 ± 9.2 (SD) μA. For the saccular ones, the average was 16.6 ± 10.5 μA. Running a simple two-tailed Welch t-test on their results provides a *p*-value of ≈ 0.025 [Sample size: saccular *n* = 43 and utricular *n* = 33 (47)]. This indicates that the increased sensitivity of the utricular nerve reported by Kushiro et al. is unlikely to be caused by sampling error (statistical term) (47). Thereby, this highly cited study paradoxically provides the direct counterevidence against the claim that the saccular projection to the SCM should be any stronger than the utricular one.

### The crossing cVEMP

The previously mentioned studies, discovering cVEMP responses to galvanic stimulation, BCV and reflex hammer taps, also uncovered the presence of an inversed myogenic potential in the SCM, contralateral to stimulation (10, 24–27). This crossing cVEMP, since denoted n12p24n30 (62), could be elicited in both healthy subjects and patients with definite unilateral vestibular loss (24, 26). Subsequently, the crossing response was also recognized as elicitable by ACS stimulation in more than 50 % of healthy subjects (10). As the saccular branch of the vestibular nerve were the only one unable to produce a contralateral inverse response in the animal studies by Uchino et al., it appears unlikely that saccular activation contributes to this crossed response. The previously mentioned threshold difference between utricular fibers and saccular ones may perhaps provide some explanation for the crossed ACS cVEMP. As with a threshold difference between the ipsilateral and contralateral ACS cVEMP in healthy humans of 12.2-13.3 dB (10, 62) they are in striking alignment with the mean inter-otolith thresholds found in animal studies (38, 39). Extending this rationale, the crossing cVEMP response is possibly driven by activation of the utricle, but a significant contribution from the SCCs may well be present (35, 40, 47). However, despite not being able to specifically pinpoint the origin of the crossing cVEMP, it provides evidence against the idea that saccular activation is the only important driver for ipsilateral cVEMP in humans.

### The theory of saccular predominance—a previous debate revisited

Animal findings presented by Curthoys et al. lead to a realization that ACS did not provide a specific saccular activation (45, 46). This challenged the fundamental basis for the clinical interpretation of cVEMP as a measure of saccular function. An alternative hypothesis was provided by Curthoys in an invited review from 2010 (6). In this review, Curthoys theorized that the ACS cVEMP must reflect saccular activation regardless of co-activation of the utricle, as “A cVEMP to ACS reflects saccular function not because it is only saccular afferents which are activated by air conducted sound, but because the SCM response which is being measured is predominantly determined by saccular activation” (6). The paucity of evidence for this hypothesis led to considerable and sometimes acrimonious debate (63–66). The crossing inverted ACS cVEMP, pointing to a non-saccular contribution, were brought forward as a principial counterargument against the simple saccular predominance theory (35, 65). However, the significance of this finding was regrettably not picked up in the replies (64, 67). Instead, the debate revolved around later published VEMP findings in patients classified as suffering from vestibular neuritis (68–70). In one of these studies, Manzari et al. reported that all patients included with a reduced inferior vestibular nerve (IVN) function showed an absent cVEMP pointing to a 100% sensitivity of the cVEMP to identify IVN loss. However, their patients with probable inferior vestibular neuritis were selected on the basis of the cVEMP’s outcome itself and not by independent criteria (70, 71). Thereby, the inclusion criteria linked the patients to the cVEMP outcomes and the conclusion that the cVEMP’s monitor saccular or IVN integrity was based on circular logic and by design only reaffirm the premises (71). So, we conclude that these studies cannot be used as clinical evidence to support the use of cVEMP’s as a measure of saccular or IVN function.

## Conclusion and future direction

More than 60 years after its discovery, there are still fundamental gaps in the understanding of what is being examined by cVEMPs. We found insufficient hard evidence in the literature for the widely spread idea that the cVEMP test can be used for testing the integrity of saccular- or inferior vestibular nerve function. As stated by Welgampola and Carey already in 2010 (35), such an interpretation still requires carefully executed lesion studies to be able to interpret the cVEMP results as such. Overall, we conclude that the cVEMP response likely reflects vestibular function through direct acoustic stimulation of hair cells within the labyrinth. Still the hypothesis that cVEMP may truly predominantly reflect the saccular or IVN function, cannot be rejected. It, however, remains unclear to which extend each organ of the vestibular labyrinth contribute to the cVEMP response in humans. It also remains a question to which extend cVEMPs represents vestibular function, as the stimulus is non-physiological. A major limitation in the validation of VEMP testing as a test of macular function is that there is still no golden standard for testing saccular or utricular function to compare with. All in all, common application and interpretation of the cVEMP as a specific test of saccular and inferior vestibular nerve integrity in clinical practice needs to be reconsidered, until more evidence is provided.

### Future direction

First, the use of cVEMP in vestibular diagnostics should be in harmony with the existing evidence, as the current clinical application of cVEMP might result in a misleading evaluation of vestibular function. Second, well-controlled studies on surgical lesions in both animal models and humans are required in order to determine whether or not the function of a single vestibular end-organ is a prerequisite for an intact cVEMP response (35). Third, extrapolation of quadrupedal animal research toward vestibular function in bipedal humans should be handled with caution (7, 72). The search of a selective stimulus for the vestibular end-organs could benefit from studies in animal models more similar to humans in terms of mechanical properties of the skull and inner ear. The findings of the ongoing research on responsiveness of the vestibular afferents to sound in primates is in this regard eagerly anticipated (40).
